# The Molecular Mechanism of Hemocyte Immune Response in *Marsupenaeus japonicus* Infected With Decapod Iridescent Virus 1

**DOI:** 10.3389/fmicb.2021.710845

**Published:** 2021-08-26

**Authors:** Zihao He, Jichen Zhao, Xieyan Chen, Minze Liao, Yuan Xue, Jianing Zhou, Haozhen Chen, Guoliang Chen, Shuang Zhang, Chengbo Sun

**Affiliations:** ^1^College of Fisheries, Guangdong Ocean University, Zhanjiang, China; ^2^Haimao Seed Technology Group Co., Ltd., Zhanjiang, China; ^3^Aquatic Animals Precision Nutrition and High Efficiency Feed Engineering Research Center of Guangdong Province, Zhanjiang, China; ^4^Guangdong Provincial Laboratory of Southern Marine Science and Engineering, Zhanjiang, China; ^5^Guangdong Provincial Key Laboratory of Pathogenic Biology and Epidemiology for Aquatic Economic Animals, Zhanjiang, China

**Keywords:** decapod iridescent virus 1, *Marsupenaeus japonicus*, enzyme activity, transcriptomic analysis, immunity response

## Abstract

As a new type of shrimp lethal virus, decapod iridescent virus 1 (DIV1) has caused huge economic losses to shrimp farmers in China. Up to now, DIV1 has been detected in a variety of shrimps, but there is no report in *Marsupenaeus japonicus*. In the current study, we calculated the LC_50_ to evaluate the toxicity of DIV1 to *M. japonicus* and determined through nested PCR that *M. japonicus* can be the host of DIV1. Through enzyme activity study, it was found that DIV1 can inhibit the activities of superoxide dismutase, catalase, lysozyme, and phenoloxidase, which could be a way for DIV1 to achieve immune evasion. In a comprehensive study on the transcriptomic changes of *M. japonicus* in response to DIV1 infection, a total of 52,287 unigenes were *de novo* assembled, and 20,342 SSR markers associated with these unigenes were obtained. Through a comparative transcriptomic analysis, 6,900 differentially expressed genes were identified, including 3,882 upregulated genes and 3,018 downregulated genes. The Gene Ontology (GO) and Kyoto Encyclopedia of Genes and Genomes (KEGG) enrichment analysis showed that some GO terms related to virus invasion, replication, and host antiviral infection were promoted under DIV1 infection, such as carbohydrate binding, chitin binding, chitin metabolic process, and DNA replication initiation, and some KEGG pathways related to immune response were significantly influenced by DIV1 infection, including Toll and IMD signaling pathway, JAK-STAT signaling pathway, IL-17 signaling pathway, C-type lectin receptor signaling pathway, complement and coagulation cascades, antigen processing and presentation, necroptosis, apoptosis, NOD-like receptor signaling pathway, apoptosis—multiple species, and TNF signaling pathway. Further analysis showed that STAT, Dorsal, Relish, heat shock protein 70 (HSP70), C-type lectins, and caspase play an important role in DIV1 infection. This is the first detailed study of DIV1 infection in *M. japonicus*, which initially reveals the molecular mechanism of DIV1 infection in *M. japonicus* by using the transcriptome analysis of hemocytes combined with enzyme activity study.

## Introduction

*Iridoviridae* is a member of a monophyletic clade of large, nucleocytoplasmic DNA viruses with double-stranded DNA genomes (İnce et al., 2018). According to their particle sizes, host range, DNA cross-hybridization, the presence of a methyltransferase, and the sequence of major capsid protein, family Iridoviridae is subclassified into five genera, including *Iridovirus*, *Chloriridovirus*, *Ranavirus*, *Lymphocystivirus*, and *Megalocytivirus* (Chinchar et al., 2017). The iridescent viruses mostly infect invertebrates and poikilothermic vertebrates, such as teleost fish, crustaceans, amphibians, insects, and reptiles (Matthews, 1979; Chinchar et al., 2011; Kurita and Nakajima, 2012; Xu et al., 2016). In recent years, Xu et al. (2016) and Qiu et al. (2017) respectively isolated and identified two iridescent viruses from red claw crayfish *Cherax quadricarinatus* and *Litopenaeus vannamei* and named them *C. quadricarinatus* iridovirus (CQIV CN01) and shrimp hemocyte iridescent virus (SHIV 20141215). Because the genome similarity between these two original isolations was 99%, the Executive Committee of the The International Committee on Taxonomy of Viruses (ICTV) (2019) identified SHIV 20141215 and CQIV CN01 as two virus isolates of decapod iridescent virus 1 (DIV1). This is the only species of the new genus *Decapodiridovirus* within the family *Iridoviridae*. As a new type of shrimp lethal virus, DIV1 has been detected in a variety of shrimps, such as *L. vannamei*, *Fenneropenaeus merguiensis*, *C. quadricarinatus*, *Exopalaemon carinicauda*, *Macrobrachium rosenbergii*, *Penaeus monodon*, and so on (Xu et al., 2016; Chen X. et al., 2019; Qiu et al., 2019; Liao X. et al., 2020; Liao X. Z. et al., 2020; He et al., 2021).

Shrimp farming is an important part of the crustacean farming industry. According to the latest worldwide statistics on aquaculture compiled by the Food and Agriculture Organization of the United Nations (FAO, 2020), the total crustacean production of the world in 2018 reached 9,386.5 thousand tonnes, with a total farmgate sale value of USD 69.3 billion. Among them, the five main cultured shrimps of *L. vannamei*, *Procambarus clarkii*, *P. monodon*, *Macrobrachium nipponense*, and *M. rosenbergii* accounted for 84.1% of the crustacean production. There is no doubt that shrimp farming had become an important source of income for crustacean farmers. However, with the rapidly increasing shrimp production, frequent outbreaks of various diseases followed. Virus pathogens, such as white spot syndrome virus (WSSV), Taura syndrome virus (TSV), and the infectious hypodermal and hematopoietic necrosis virus (IHHNV), still represent one of the major impediments to the development of shrimp culture (Thitamadee et al., 2016; Li C. et al., 2019). In recent years, the newly discovered shrimp lethal virus DIV1 has brought a huge challenge to the shrimp aquaculture industry due to its wide host spectrum and strong toxicity. On the one hand, the wide host spectrum makes DIV1 widely existent in natural and breeding environments, which makes it difficult for people to effectively control its spread. On the other hand, the strong toxicity of DIV1 can cause the large-scale deaths of farmed shrimps and cause huge economic losses to farmers. Kuruma shrimp *Marsupenaeus japonicus* is widely distributed in the Indo-Western Pacific region and the East and South China seas (Dall et al., 1990). Because of its high economic value, *M. japonicus* has become one of the main cultured shrimp species in China. Only in 2019, 55,228 tonnes of *M. japonicus* was harvested in China, which brought substantial economic benefits to the shrimp farmers (FBMA, 2020). To date, there are no detailed reports on DIV1 infection of *M. japonicus*.

Similar to other invertebrates, crustaceans mainly rely on their innate immune system, consisting of humoral immunity and cellular immunity, to defend against pathogens (Li and Xiang, 2013). Hemocytes are a major part of the shrimp innate immune system, which not only remove foreign substances through phagocytosis, encapsulation, and nodule formation but also resist pathogens and repair cell damage by producing reactive oxygen species (ROS), antimicrobial peptides, immune enzymes, and other immune factors (Jiravanichpaisal et al., 2006; Cerenius et al., 2010). Superoxide dismutase (SOD), catalase (CAT), lysozyme (LYZ), and phenoloxidase (PO) are four important immune enzymes, which have the function of anti-oxidant and anti-pathogen (Holmblad and Söderhäll, 1999; Cerenius and Söderhäll, 2004; Kaizu et al., 2011). The activity of these immune enzymes is usually used to reflect the impact of shrimp immunity after a pathogen infection (such as WSSV, IHHNV, *Vibrio parahaemolyticus*, and *Spiroplasma eriocheiris*) (Arockiaraj et al., 2012; Ren et al., 2020; Zhu et al., 2020; Jiao et al., 2021), harmful substance invasion (such as microcystin and tributyltin) (Wu et al., 2014; Chen et al., 2015), or dietary improvement (such as polypeptides and dietary hydrolyzable tannins) (Liao et al., 2019; Zhu et al., 2021). With the rapid development of high-throughput RNA sequencing technologies, including RNA sequencing (RNA-seq), more and more studies use RNA-seq to expose the immune molecular mechanism of shrimp against viral infections, bacterial infections, and environmental stress (Kurita and Nakajima, 2012; Li and Xiang, 2013; Wang et al., 2014; Zeng et al., 2014; Qin et al., 2018; Jin and Zhu, 2019; Nian et al., 2019; Duan et al., 2020). Among them, hemocytes were widely used in transcriptome studies of shrimp virus infections, such as WSSV, TSV, and DIV1 (Sookruksawong et al., 2013; Ghani and Bhassu, 2019; Wang et al., 2019; Liao X. et al., 2020). Therefore, the immune molecular mechanism of shrimp against a virus infection can be better understood by using the transcriptome analysis of hemocytes combined with enzyme activity study.

Since there is no detailed report on the DIV1 infection of *M. japonicus*, RNA-seq was applied in this study to explore the molecular mechanisms of *M. japonicus* hemocytes against DIV1 infection, based on the results of the LC_50_ test and the enzyme activity analysis. The result is of great value for diagnosing shrimp DIV1 infection and developing methods to prevent virus outbreaks.

## Materials and Methods

### Shrimp Culture

The study protocol was approved by the Ethics Review Board of the Institutional Animal Care and Use Committee in Guangdong Ocean University. Healthy juvenile *M. japonicus* were purchased from Suixi County Zhongli Aquatic Products Co., Ltd., and raised in the East Island Marine Biology Research Base, Guangdong Ocean University in Zhanjiang, Zhanjiang, China. Before the LC_50_ test, every 30 healthy *M. japonicus* (10.5 ± 1.6 g) were randomly selected to acclimatize for 1 week in a 0.3-m^3^ tanks with filtered seawater and continuous aeration. The salinity, pH, and temperature of the seawater were maintained at ∼30‰, ∼7.5, and ∼28 C, respectively. The shrimp were fed three times per day with commercial shrimp expanded pellets (Tongwei Co., Ltd., China), and the seawater was changed nearly 90% once a day. The shrimp were then randomly sampled and examined for potential pathogens by PCR to ensure that they were free from WSSV, IHHNV, and DIV1. The primers for pathogen detection are shown in Table 1.

**TABLE 1:** Primers used in the validation of gene expression.

No.	Primer names	Sequences(5′–3′)
	Nested PCR	
1	DIV1-F1	GGGCGGGAGATGGTGTTAGAT
2	DIV1-R1	TCGTTTCGGTACGAAGATGTA
3	DIV1-F2	CGGGAAACGATTCGTATTGGG
4	DIV1-R2	TTGCTTGATCGGCATCCTTGA
	qPCR	
5	qRT-DIV1-F	TCGTTTCGGTACGAAGATGTA
6	qRT-DIV1-R	TTTCACACTTCCTGATAGTCTTCCAT
7	TaqMan probe	TCACAGAAAAGATTCCCGAAATGGTAAAAC
8	TPS-F	ACGACATCTCGCTGCTCAA
9	TPS-R	TGGGAACGGTCACCTTCAT
10	CLEC2-F	AAGCGACTTCTGGATTGGA
11	CLEC2-R	CTAGAGGCATGGGAGTGTCA
12	RNF152-F	TTGTCAGTAGCCTCTGGTGC
13	RNF152-R	GCGGTGGAATAAGTGGTGTC
14	Hsp70-F	TGCTTCACCATCAAATCCTC
15	Hsp70-R	CGTCTGTTCCAATTCCTTCA
16	proPO-F	AACGGGGTATCCTTCTGTGG
17	proPO-R	ATAGTCTGCGGCATCTTCG
18	Crus4-F	CCTGCTCCAACGACTACAAG
19	Crus4-R	GAGGTTTCCCAAAGACTGATG
20	Cu/Zn-SODi5-F	ACAGGGAACATCACGGGACT
21	Cu/Zn-SODi5-R	CAGGATCTCAACGTAAGCGAC
24	PPAE2-F	CCTCCTTCTATCGCTACGG
25	PPAE2-R	CATCAGGCTTTCCTTCCAC
26	CLYZ-F	CCTCCTGGACGACGACTTG
27	CLYZ-R	ACGTATGCGACCCATGCTG
28	STAT-F	TCCGTCGGGTCCAAGGTAT
29	STAT-R	CGAGGGACTGGGCATACTG
30	Dorsal-F	ACCACCAACATAATAAGAAACC
31	Dorsal-R	ATGACACCAGGAGGAGCAG
32	Relish-F	GCACAACTTCGCAAACCAC
33	Relish-R	TGCCTCTTCTTCAGCCTCC
34	EF1-α-F	GGAACTGGAGGCAGGACC
35	EF1-α-R	AGCCACCGTTTGCTTCAT

### LC_50_ Test

In the LC_50_ test, *M. japonicus* were randomly divided into seven groups; each group had three tanks. Among them, each healthy *M. japonicus* in six groups was intramuscularly injected with 50 μl of viral inoculum, and the concentrations were 3.95 × 10^9^, 3.95 × 10^8^, 3.95 × 10^7^, 3.95 × 10^6^, 3.95 × 10^5^, and 3.95 × 10^4^ copies/μg DNA, respectively. The other group was injected with 50 μl phosphate-buffered saline (PBS, pH 7.4) instead of the viral inoculum. The cumulative survival rate was counted every 4 h, and dead shrimp were picked up to avoid a secondary infection. The methods of viral inoculum preparation and quantification can be found in previous studies (Qiu et al., 2018; Chen X. et al., 2019). The DIV1 inoculum was tested by nest PCR to ensure that it was not contaminated with WSSV and IHHNV, and the nest PCR primers, specific qPCR primers, and TaqMan probe for DIV1 detection and quantification are shown in Table 1. The LC_50_ was calculated by probit analysis on SPSS 19.0 program (SPSS Inc., Chicago, IL, United States) using the Bliss method (Bliss, 1939).

### Sample Collection

A total of 30 healthy *M. japonicus* injected with 50 μl of DIV1 inoculum of 3.95 × 10^9^ copies/μg DNA were used as the DIV1-infected group, and the other 30 healthy *M. japonicus* injected with 50 μl of PBS were used as the negative control group. The weight of *M. japonicus* and the culture conditions were also kept the same as in the LC_50_ test (refer to the previous article; He et al., 2021). The *M. japonicus* at 24 h post-injection (hpi) were selected to make samples under an aseptic condition. The hemolymph was withdrawn into modified ACD anticoagulant solution, and the hemocytes and plasma were separated by centrifugation (3,000 × *g* for 5 min at 4 C). The plasma and hemocytes from three individuals in the same group were combined as one sample and immediately frozen in liquid nitrogen before storing at −80 C until enzyme activity analysis or RNA extraction.

### Enzyme Activity Analysis

Prior to analysis, the homogenized solid samples were homogenized in pre-chilled PBS (1:9 dilution) and then centrifuged for 10 min (4 C and 5,000 × *g*) to obtain the supernatant for further use. SOD, CAT, LYZ, and PO in the plasma were measured using commercial detection kits (Jianglai Bioengineering Institute, Shanghai, China) according to the protocols of the manufacturer.

### RNA Extraction and Transcriptome Sequencing

Total RNA was extracted separately from the hemocytes of three non-infected and three DIV1-infected samples using TranZol Up Plus RNA Kit (TransGen, Beijing, China) following the protocol of the manufacturer, and the RNA concentration was determined through SimpliNano (GE Healthcare, United States). Fragmentation buffer was used to break the mRNA into short fragments. Using mRNA as a template, the first-strand cDNA was synthesized using random hexamers, and then buffer, dNTPs, RNase H, and DNA polymerase I were added to synthesize the second-strand cDNA. After purification and elution with EB buffer, cDNA end-repair, and adenylation at the 3′ end, poly (A) was added and the sequencing adaptor was connected. Finally, transcriptome sequencing was performed by BGI (Shenzhen, China) with the Illumina Genome Analyzer technology.

### *De novo* Assembly and Data Annotation

Raw reads were filtered to remove adaptor sequences, ambiguous “N” nucleotides (with a ratio of “N” more than 5%), and low-quality sequences (with a quality score less than 15) using trimmomatic software (v0.36) (Bolger et al., 2014). Meanwhile, the Q20, Q30, and GC contents of the clean reads were calculated. *De novo* assembly was accomplished using Trinity software (v2.0.6) (Haas et al., 2013). Based on the sequence similarity and length, TGICL (v2.1) was used to remove redundant sequences and generate unigenes (Pertea et al., 2003). The completeness of the assembly was assessed using BUSCO (v3.0.2) with the BUSCO arthropod dataset (Seppey et al., 2019). The assembled unigenes were annotated with five functional databases, including Kyoto Encyclopedia of Genes and Genomes (KEGG),^1^ Gene Ontology (GO),^2^ Nr,^3^ Swiss-Prot,^4^ and Clusters of Orthologous Genes.^5^

### Analysis of Differentially Expressed Genes and Functional Enrichment

Fragments per kilobase of transcript per million reads method was used to measure the transcript expression levels of the unigenes. A differential expression analysis of the two groups was performed using the DESeq2 package (1.30.0). The *p*-value was adjusted using the *q*-value, and genes with *q*-value < 0.05 and | log_2_ (fold change)| > 1 found by DESeq2 were considered as differentially expressed genes (DEGs) (Love et al., 2014). All DEGs were further analyzed using GO and KEGG database to look for significantly enriched GO and KEGG terms.

### Identification of Simple Sequence Repeat Loci in the Transcripts

SSR molecular marker technology has been wildly applied in the field of aquatic breeding, inheritance, and evolution (Andriantahina et al., 2013; Wang et al., 2018; Huang et al., 2020). To identify potential SSR loci from the transcriptome, sequences with six types of repeat unit, including mono-nucleotide, di-nucleotide, tri-nucleotide, tetra-nucleotide, penta-nucleotide, and hexa-nucleotide repeat SSR, were detected using the MISA software.^6^ The parameters were set for the detection of mono-nucleotide, di-nucleotide, tri-nucleotide, tetra-nucleotide, penta-nucleotide, and hexa-nucleotide repeat SSR with a minimum of 12, six, five, five, four, and four repetitive units, respectively (Wei et al., 2017).

### Validation by qRT-PCR

To validate the gene expression profiles from Illumina sequencing results, five upregulated DEGs and seven downregulated DEGs were selected to perform qRT-PCR. Among them were STAT, Dorsal and Relish belonging to JAK-STAT, Toll, and IMD signaling pathway, which are very important for the host against viral infection. Primer 5.0 software was used to design the primers for these DEGs based on the sequences obtained by Illumina sequencing, and *elongation factor-1 gene alpha* (*EF-1*α) of *M. japonicus* was used as an internal reference gene. The specific primers are shown in Table 1. The qRT-PCR was performed using Bio-Rad CFX-96 real-time PCR system. The template cDNA was reverse-transcribed using the 5× All-in-One RT Master Mix (Applied Biological Materials, Vancouver, BC, Canada) according to the protocol of the manufacturer. The qRT-PCR reaction was performed with a 20-μl reaction mixture containing 10 μl of 2× ChamQ Universal SYBR qPCR Master Mix, 1 μl of each primer (10 μM), 2 μl of diluted cDNA template, and 7 μl of ultrapure water. The reaction cycle parameters were as follows: 95°C for 2 min, 40 cycles of 95°C for 15 s, and 60°C for 30 s. All reactions were performed with three technical replicates. The relative expression ratio of the target gene *versus EF-1*α was calculated by the 2^–ΔΔCt^ method (Livak and Schmittgen, 2001).

## Results

### LC_50_ of DIV1 for *M. japonicus*

**FIGURE 1:**
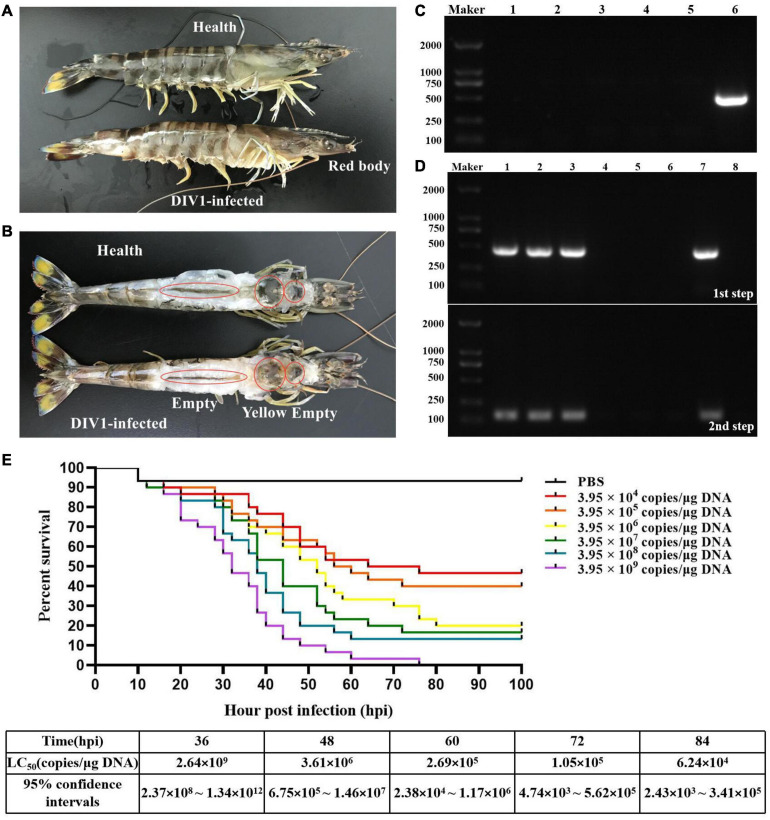
Clinical symptoms and LC_50_ test. **(A,B)** Clinical symptoms of DIV1-infected *Marsupenaeus japonicus*. **(C)** Virus detection of healthy *M. japonicus* and diseased shrimp used for LC_50_ test. Marker, DL2000 molecular mass marker; lanes 1–3: PCR-amplified products used for WSSV, IHHNV, and DIV1 detection in healthy *M. japonicus*; lanes 4–6, PCR-amplified products used for WSSV, IHHNV, and DIV1 detection in diseased shrimp. **(D)** DIV1 detection of *M. japonicus* hemocytes using nested PCR method. Marker: DL2000 molecular mass marker; lanes 1-3: PCR amplified products in DIV1-infected group; lanes 4-6: PCR amplified products in negative control group; lanes 7 and 8: PCR amplified products in positive and negative controls. **(E)** Seven groups of healthy *M. japonicus* were intramuscularly injected with 50 μl of DIV1 inoculum at six concentrations and phosphate-buffered saline as a control.

*Marsupenaeus japonicus* infected with DIV1 showed obvious clinical symptoms, including empty stomach and intestine, atrophy of the hepatopancreas with yellowing, red body, and soft shell (Figures 1A,B). As shown in Figures 1C,D, only DIV1 was detected in the diseased *M. japonicus* used for the DIV1 inoculum and the *M. japonicus* injected with DIV1. The survival rates of *M. japonicus* after exposure to DIV1 at different doses are shown in Figure 1E. The shrimp mortality rate increased as the virus dose increased. *M. japonicus* infected with 3.95 × 10^9^ copies/μg DNA DIV1 inoculum had a mortality rate of 100% at 76 hpi, and the mortality rate of the other injection doses was stable at 80 hpi. Probit analysis showed that the LC_50_ of DIV1 infection in *M. japonicus* is 2.64 × 10^9^, 3.61 × 10^6^, 2.69 × 10^5^, 1.05 × 10^5^, and 6.24 × 10^4^ copies/μg DNA at 36, 48, 60, 72, and 84 hpi, respectively (Figure 1E).

### Immune Enzyme Activities in the Tissues of *M. japonicus*

**FIGURE 2:**
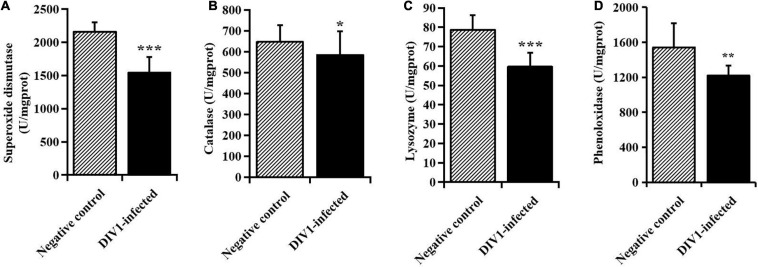
The activities of immune enzymes in the plasma of *Marsupenaeus japonicus* (mean ± SD). **(A)** Superoxide dismutase, **(B)** catalase, **(C)** lysozyme, and **(D)** phenoloxidase (PO). The statistically significant differences between the two groups were calculated by Student’s *t*-test (*p < 0.05, **p < 0.01, ***p < 0.001).

After infection with DIV1 at 24 hpi, the activities of SOD, CAT, LYZ, and PO in the plasma were detected to evaluate the effect of DIV1 infection on the immune enzymes of *M. japonicus* (Figure 2). The results showed that the activity of SOD in the plasma was the highest, followed by PO, CAT, and LYZ. It was worth noting that, after infection with DIV1, the activities of the four immune enzymes were all significantly decreased in the plasma (*p* < 0.05).

### Statistics of Transcriptome Sequencing and *de novo* Assembly

**FIGURE 3:**
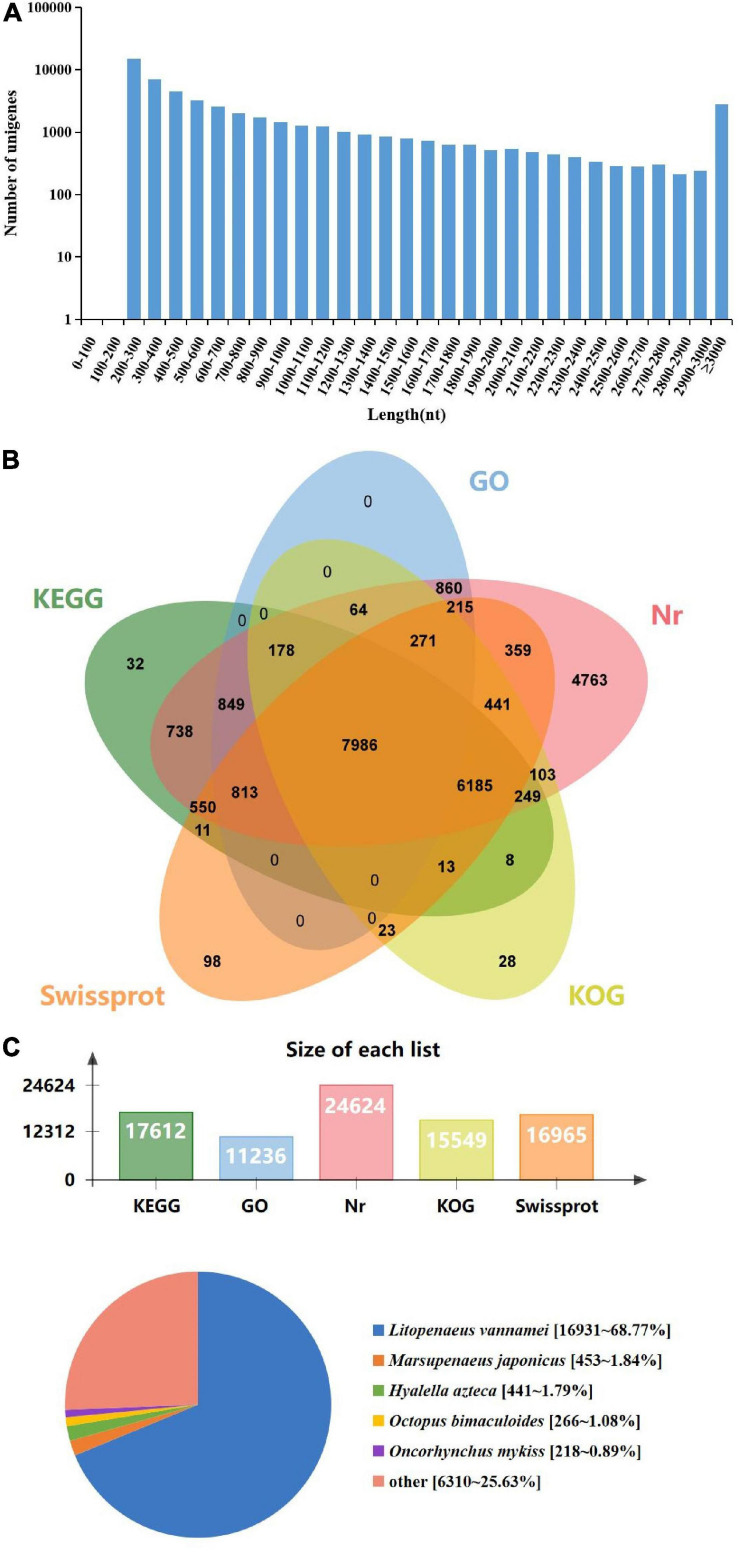
Length distribution, annotation, and species distribution of transcriptome unigenes. **(A)** The *X*-axis indicates the length of the unigenes, while the *Y*-axis indicates the number of unigenes. **(B)** The Venn diagram shows the annotation of the unigenes from the *Marsupenaeus japonicus* transcriptome. **(C)** The species distribution of unigene BLASTx matches against the Nr protein database and the proportions for each species.

To identify immune-related genes which are vital for *M. japonicus* defense against DIV1 infection, six cDNA libraries were prepared from hemocyte samples collected from DIV1-infected and non-infected *M. japonicus* with three biological replicates. After the removal of the adapter sequence and low-quality reads, a total of 127,748,656 and 128,230,224 clean reads that represent a total of 19.16 and 19.24 Gb nucleotides were generated for the negative control and DIV1-infected group, respectively. The CG content of the clean reads was 39.63% in the negative control group and 40.04% in the DIV1-infected group, respectively (Table 2). All sequencing reads were deposited into the Sequence Read Archive of the National Center for Biotechnology Information and are available with the accession number PRJNA720475.^7^ After removing redundancy of the assembled contigs, a total of 52,287 unigenes were recovered. The size and the length distribution of the negative control group and the DIV1-inected group unigenes are shown in Figure 3A. Among them, most of the unigenes (14,968, 28.63%) were 200–300 nt in length, followed by 300–400 nt (6,984, 13.36%), and 2,773 unigenes (8.66%) were ≥ 3,000 nt.

**TABLE 2:** Summary of clean reads statistics by Illumina sequencing.

Samples	Control	DIV1-infected
Raw reads number	131,463,150	131,463,150
Clean reads number	127,748,656	128,230,224
Clean bases (Gb)	19.16	19.24
Q20 (%)	97.21	97.18
Q30 (%)	92.89	92.81
Clean reads ratio (%)	97.17	97.54
GC (%)	39.63	40.04

### Annotation of the Assembled Sequences

To obtain comprehensive function information of unigenes, the unigenes were annotated in five major databases (Figure 3B). These databases included KEGG (17,612 unigenes), GO (11,236 unigenes), Nr (24,624 unigenes), Eukaryotic Orthologous Groups (KOG; 15,549 unigenes), and Swiss-Prot (16,965 unigenes). The analysis showed that among the 52,287 unigenes, most of the unigenes were annotated in Nr. The species distribution of the most significant hits in the Nr database was examined to learn the sequence conservation of *M. japonicus* compared with other species (Figure 3C). Over 74% of the total unigenes matched with the sequences from five top-hit species: *L. vannamei* (68.77%), *M. japonicus* (1.84%), *Hyalella azteca* (1.79%), *Octopus bimaculoides* (1.08%), and *Oncorhynchus mykiss* (0.89%), all of which belong to aquatic organisms.

**FIGURE 4:**
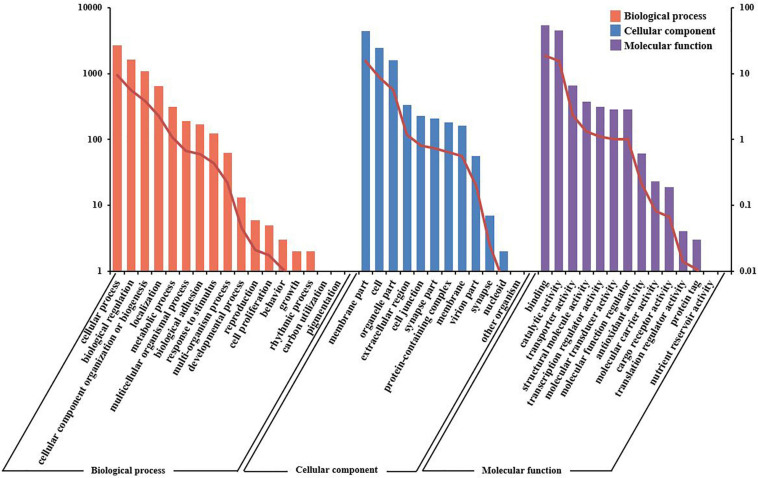
Gene ontology (GO) terms and annotation of the integrated transcriptome assembly. Three main GO categories: cellular component, molecular function, and biological process. The *X*-axis indicates GO categories, while the *Y*-axis indicates the number of genes and percentage of genes.

The unigenes were aligned to GO terms; 28,444 unigenes were mainly divided into three categories with 42 functional groups: biological process (17 functional groups), cellular component (12 functional groups), and molecular function (13 functional groups). In the biological process category, most unigenes were involved in the “cellular process” (9.40%), “biological regulation” (5.70%), and “cellular component organization or biogenesis” (3.80%). In the category of cellular component, “membrane part” (15.56%) and “cell” (8.59%) were the most represented. As for the molecular function category, “binding” (18.92%) and “catalytic activity” (15.77%) were the dominant groups (Figure 4).

**FIGURE 5:**
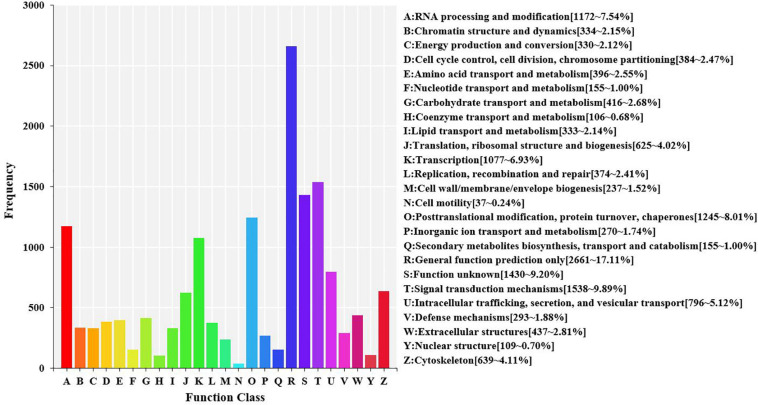
Eukaryotic orthologous groups (KOG) function classification in the transcriptome of hemocytes in *Marsupenaeus japonicus* hemocytes. Each bar represents the number of unigenes classified into each of the 25 KOG functional categories. The *X*-axis stands for the functional categories, and the *Y*-axis indicates the frequency.

Using the KOG database to further explore the protein orthologs of the assembled unigenes, 15,549 unigenes were successfully annotated with 25 specific protein function definitions or orthologous categories. The largest three categories were “general function prediction only” (2,661, 17.11%), “signal transduction mechanisms” (1,538, 9.89%), and “function unknown” (1,430, 9.20%). The two smallest categories were “coenzyme transport and metabolism” (106, 0.68%) and “cell motility” (37, 0.24%) (Figure 5).

**FIGURE 6:**
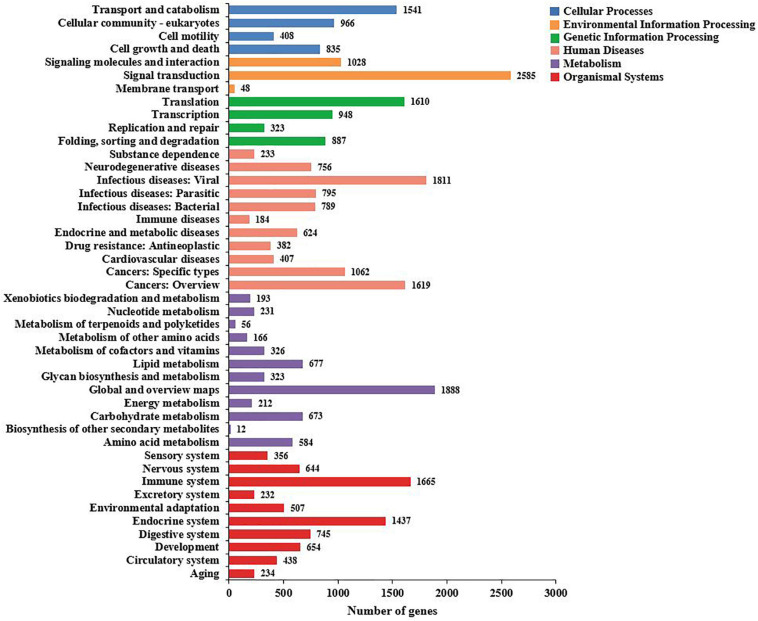
The Kyoto Encyclopedia of Genes and Genomes (KEGG) biological pathway classification histograms for annotated unigenes identified in the hemocytes of *Marsupenaeus japonicus*. Each bar represents the number of unigenes classified into different biological processes. The *X*-axis shows the number of matched unigenes, while the *Y*-axis shows the pathways from the KEGG classification.

To identify the biological processes of the unigenes, 32,094 unigenes that were assigned were annotated using the KEGG database and assigned to different pathways in six major groups of the KEGG pathways, including cellular processes, environmental information processing, genetic information processing, human diseases, metabolism, and organismal systems. These annotated unigenes were further divided into 44 levels and two subcategories. The largest subcategory group was signal transduction (2,585 unigenes), followed by global and overview maps (1,888 unigenes) and infectious diseases: viral (2,304 unigenes) (Figure 6).

### Identification and Functional Characterization of DEGs

**FIGURE 7:**
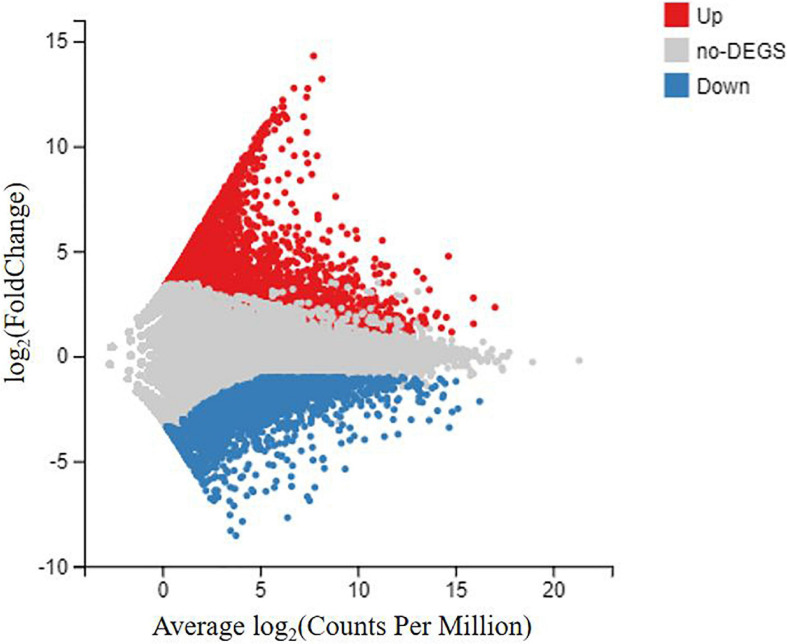
MA plot between differentially expressed genes (DEGs) of the DIV1-infected group and the negative control group of *Marsupenaeus japonicus* hemocytes. The *X*-axis indicates the average expression level, and the *Y*-axis indicates the fold change. Red dots represent the significantly upregulated DEGs, while blue dots represent the significantly downregulated DEGs [*q*-value < 0.05 and | log_2_ (fold change)| > 1]. Gray dots represent the DEGs which were not significantly different.

To identify the DEGs between the DIV1-infected group and the negative control group, the transcript abundance in each unigene was compared between the two groups using the FRKM approach. A total of 6,900 DEGs were screened, including 3,882 upregulated genes and 3,018 downregulated genes, with a cut-off *q*-value < 0.05 and | log_2_ (fold change)| > 1 (Figure 7). The DEGs were found to have a variety of biological functions according to the Nr annotation. Among them, some well-characterized genes implicated in innate immune defense, such as Dorsal, Relish, C-type lectin 1 (CTL1), caspase 1, inhibitor of apoptosis protein, heat shock protein 70 (HSP70), heat shock protein 90, and prophenoloxidase activating enzyme 2 (PPAE2) (Table 3).

**TABLE 3:** Candidate genes involved in *M. japonicus* immune response against DIV1.

Category or gene ID	Homologs function	Species	log_2_FC
**Toll and IMD signaling pathway**			
CL5243.Contig17_All	Ankyrin-1-like	*Litopenaeus vannamei*	2.66
Unigene32575_All	Ankyrin-3-like	*Litopenaeus vannamei*	4.94
CL1473.Contig2_All	Caspase-2-like isoform X1	*Litopenaeus vannamei*	3.71
CL1473.Contig3_All	Caspase-2-like isoform X1	*Litopenaeus vannamei*	2.96
CL1210.Contig2_All	Clotting factor B-like	*Litopenaeus vannamei*	−2.53
CL1130.Contig3_All	Dorsal	*Marsupenaeus japonicus*	1.26
Unigene59_All	Dual oxidase	*Marsupenaeus japonicus*	−1.76
CL661.Contig2_All	Immune deficiency homolog	*Marsupenaeus japonicus*	3.02
CL2527.Contig3_All	Phenoloxidase-activating factor 3-like	*Litopenaeus vannamei*	1.31
CL21.Contig3_All	Prophenoloxidase activating enzyme 2	*Litopenaeus vannamei*	−3.03
CL2652.Contig4_All	Prophenoloxidase activating enzyme 2a	*Penaeus monodon*	−3.36
Unigene4922_All	NF-κB transcription factor Relish	*Litopenaeus vannamei*	2.33
CL1572.Contig2_All	Relish	*Litopenaeus vannamei*	1.87
CL3550.Contig3_All	Serine proteinase	*Penaeus chinensis*	−3.37
CL2544.Contig1_All	Transcription factor ATF-b	*Litopenaeus vannamei*	2.74
Unigene21552_All	Tryptase-like	*Litopenaeus vannamei*	5.53
Unigene17584_All	Ubiquitin-conjugating enzyme E2	*Penaeus chinensis*	5.55
**IL-17 signaling pathway**			
CL4327.Contig2_All	Caspase-1-like	*Litopenaeus vannamei*	1.56
CL1473.Contig2_All	Caspase-2-like isoform X1	*Litopenaeus vannamei*	3.71
CL1473.Contig3_All	Caspase-2-like isoform X1	*Litopenaeus vannamei*	2.96
CL4401.Contig2_All	Hemocytin	*Litopenaeus vannamei*	−3.87
CL3865.Contig1_All	Heat shock protein 90	*Penaeus monodon*	3.23
Unigene12055_All	Heat shock protein 90	*Penaeus monodon*	2.21
Unigene332_All	Heat shock protein 90	*Penaeus monodon*	2.65
CL3336.Contig3_All	Interferon alpha-inducible protein 27-like protein 2B	*Litopenaeus vannamei*	4.38
Unigene8433_All	Macrophage mannose receptor 1-like	*Litopenaeus vannamei*	5.33
Unigene17910_All	Mucin-12	*Litopenaeus vannamei*	1.44
Unigene4817_All	Mucin-2-like	*Maylandia zebra*	2.79
Unigene3453_All	Mucin-5AC-like	*Oncorhynchus kisutch*	1.89
Unigene4922_All	NF-κB transcription factor Relish	*Litopenaeus vannamei*	2.33
CL1572.Contig2_All	Relish	*Litopenaeus vannamei*	1.87
Unigene12839_All	Zinc proteinase	*Astacus astacus*	6.73
CL2859.Contig2_All	Zinc proteinase Mpc1	*Litopenaeus vannamei*	8.22
**C-type lectin receptor signaling pathway**			
CL1473.Contig2_All	Caspase-2-like isoform X1	*Litopenaeus vannamei*	3.71
CL1473.Contig3_All	Caspase-2-like isoform X1	*Litopenaeus vannamei*	2.96
Unigene10996_All	C-type lectin 1	*Marsupenaeus japonicus*	4.61
Unigene31896_All	C-type lectin domain family 4 member E-like	*Litopenaeus vannamei*	3.46
Unigene4128_All	C-type lectin domain family 6 member A-like	*Litopenaeus vannamei*	8.10
CL2041.Contig1_All	Cyclooxygenase	*Marsupenaeus japonicus*	4.89
Unigene3777_All	Lectin A isoform 2, partial	*Marsupenaeus japonicus*	5.48
Unigene6201_All	Lectin D, partial	*Marsupenaeus japonicus*	6.09
Unigene3926_All	Lectin E	*Marsupenaeus japonicus*	9.32
Unigene4922_All	NF-κB transcription factor Relish	*Litopenaeus vannamei*	2.33
CL2041.Contig8_All	Prostaglandin G/H synthase 2-like	*Litopenaeus vannamei*	3.31
Unigene20653_All	Ras protein	*Marsupenaeus japonicus*	2.63
CL1572.Contig2_All	Relish	*Litopenaeus vannamei*	1.87
CL3887.Contig2_All	Tyrosine-protein kinase Src64B-like	*Litopenaeus vannamei*	1.34
Unigene13512_All	Tyrosine-protein kinase SRK3-like	*Litopenaeus vannamei*	2.41
**Complement and coagulation cascades**			
Unigene2559_All	Alpha 2 macroglobulin	*Litopenaeus vannamei*	−4.01
CL538.Contig3_All	Coagulation factor V	*Tupaia chinensis*	−1.87
Unigene6800_All	Hemocyte transglutaminase	*Litopenaeus vannamei*	−4.31
CL4401.Contig2_All	Hemocytin	*Litopenaeus vannamei*	−3.87
Unigene2375_All	Macroglobulin	*Palaemon carinicauda*	−3.99
Unigene4488_All	Neurotrypsin-like	*Litopenaeus vannamei*	3.95
CL2265.Contig3_All	Trypsin	*Marsupenaeus japonicus*	8.98
Unigene21552_All	Tryptase-like	*Litopenaeus vannamei*	5.53
**Antigen processing and presentation**			
Unigene2969_All	Calnexin	*Marsupenaeus japonicus*	1.25
CL1414.Contig1_All	Calreticulin precursor	*Penaeus chinensis*	1.91
CL2524.Contig2_All	Cathepsin L	*Marsupenaeus japonicus*	8.04
CL4263.Contig2_All	Gamma-interferon-inducible lysosomal thiol reductase	*Penaeus merguiensis*	3.73
Unigene14001_All	Heat shock protein	*Cherax destructor*	4.56
Unigene14527_All	Heat shock protein 70	*Litopenaeus vannamei*	5.27
CL3865.Contig1_All	Heat shock protein 90	*Penaeus monodon*	3.23
Unigene12055_All	Heat shock protein 90	*Penaeus monodon*	2.21
Unigene332_All	Heat shock protein 90	*Penaeus monodon*	2.65
CL1625.Contig4_All	Legumain-like	*Litopenaeus vannamei*	7.77
**Necroptosis**			
CL1473.Contig2_All	Caspase-2-like isoform X1	*Litopenaeus vannamei*	3.71
CL1473.Contig3_All	Caspase-2-like isoform X1	*Litopenaeus vannamei*	2.96
Unigene14217_All	Ferritin subunit-like	*Litopenaeus vannamei*	1.14
CL590.Contig1_All	Glutamate dehydrogenase	*Litopenaeus vannamei*	−1.75
Unigene21356_All	Glutamine synthetase	*Marsupenaeus japonicus*	7.01
CL3865.Contig1_All	Heat shock protein 90	*Penaeus monodon*	3.23
Unigene12055_All	Heat shock protein 90	*Penaeus monodon*	2.21
Unigene332_All	Heat shock protein 90	*Penaeus monodon*	2.65
CL4255.Contig2_All	Histone H2A-like	*Zootermopsis nevadensis*	2.79
Unigene9938_All	Histone H2A-like	*Litopenaeus vannamei*	1.82
CL2425.Contig1_All	Inhibitor of apoptosis protein	*Penaeus monodon*	1.54
Unigene17549_All	Sphingomyelin phosphodiesterase	*Litopenaeus vannamei*	5.14
**Apoptosis**			
CL2922.Contig1_All	Caspase 1	*Marsupenaeus japonicus*	1.23
CL5308.Contig2_All	Caspase 1	*Marsupenaeus japonicus*	2.69
CL5308.Contig4_All	Caspase 1	*Marsupenaeus japonicus*	1.05
CL3003.Contig2_All	Caspase-1-like	*Litopenaeus vannamei*	2.87
CL4327.Contig2_All	Caspase-1-like	*Litopenaeus vannamei*	1.56
CL1473.Contig2_All	Caspase-2-like isoform X1	*Litopenaeus vannamei*	3.71
CL1473.Contig3_All	Caspase-2-like isoform X1	*Litopenaeus vannamei*	2.96
CL2473.Contig2_All	Cathepsin L	*Litopenaeus vannamei*	1.30
CL2524.Contig1_All	Cathepsin L	*Marsupenaeus japonicus*	7.79
CL2524.Contig2_All	Cathepsin L	*Marsupenaeus japonicus*	8.04
CL2524.Contig2_All	Cathepsin L	*Marsupenaeus japonicus*	8.04
Unigene2905_All	Cytochrome c isoform X1	*Litopenaeus vannamei*	1.84
Unigene10015_All	Cytochrome c isoform X2	*Litopenaeus vannamei*	1.83
Unigene2446_All	E3 ubiquitin-protein ligase RBBP6-like	*Litopenaeus vannamei*	1.29
CL2425.Contig1_All	Inhibitor of apoptosis protein	*Penaeus monodon*	1.54
CL646.Contig1_All	Lysosomal aspartic protease-like	*Litopenaeus vannamei*	6.86
Unigene4922_All	NF-κB transcription factor Relish	*Litopenaeus vannamei*	2.33
CL1572.Contig2_All	Relish	*Litopenaeus vannamei*	1.87
CL2544.Contig1_All	Transcription factor ATF-b	*Litopenaeus vannamei*	2.74
Unigene16368_All	Transcription factor ATF-b	*Litopenaeus vannamei*	6.72
Unigene2591_All	Tumor suppressor protein p53	*Macrobrachium olfersii*	−2.10
**NOD-like receptor signaling pathway**			
CL112.Contig1_All	Beta-arrestin 2	*Marsupenaeus japonicus*	1.40
CL1473.Contig2_All	Caspase-2-like isoform X1	*Litopenaeus vannamei*	3.71
CL1473.Contig3_All	Caspase-2-like isoform X1	*Litopenaeus vannamei*	2.96
CL3865.Contig1_All	Heat shock protein 90	*Penaeus monodon*	3.23
Unigene12055_All	Hsp90	*Penaeus monodon*	2.21
Unigene332_All	Hsp90	*Penaeus monodon*	2.65
CL2425.Contig1_All	Inhibitor of apoptosis protein	*Penaeus monodon*	1.54
Unigene4922_All	NF-κB transcription factor Relish	*Litopenaeus vannamei*	2.33
CL1572.Contig2_All	Relish	*Litopenaeus vannamei*	1.87
Unigene9575_All	Thioredoxin-2-like	*Litopenaeus vannamei*	1.39
**Apoptosis – multiple species**			
CL2922.Contig1_All	Caspase 1	*Marsupenaeus japonicus*	1.23
CL5308.Contig2_All	Caspase 1	*Marsupenaeus japonicus*	2.69
CL5308.Contig4_All	Caspase 1	*Marsupenaeus japonicus*	1.05
CL3003.Contig2_All	Caspase-1-like	*Litopenaeus vannamei*	2.87
CL4327.Contig2_All	Caspase-1-like	*Litopenaeus vannamei*	1.56
CL1473.Contig2_All	Caspase-2-like isoform X1	*Litopenaeus vannamei*	3.71
CL1473.Contig3_All	Caspase-2-like isoform X1	*Litopenaeus vannamei*	2.96
CL524.Contig3_All	Caspase-3-like	*Litopenaeus vannamei*	5.97
Unigene2905_All	Cytochrome c isoform X1	*Litopenaeus vannamei*	1.84
Unigene10015_All	Cytochrome c isoform X2	*Litopenaeus vannamei*	1.83
CL2425.Contig1_All	Inhibitor of apoptosis protein	*Penaeus monodon*	1.54
**TNF signaling pathway**			
CL2922.Contig1_All	Caspase 1	*Marsupenaeus japonicus*	1.23
CL5308.Contig2_All	Caspase 1	*Marsupenaeus japonicus*	2.69
CL5308.Contig4_All	Caspase 1	*Marsupenaeus japonicus*	1.05
CL3003.Contig2_All	Caspase-1-like	*Litopenaeus vannamei*	2.87
CL4327.Contig2_All	Caspase-1-like	*Litopenaeus vannamei*	1.56
CL1473.Contig2_All	Caspase-2-like isoform X1	*Litopenaeus vannamei*	3.71
CL1473.Contig3_All	Caspase-2-like isoform X1	*Litopenaeus vannamei*	2.96
CL2041.Contig1_All	Cyclooxygenase	*Marsupenaeus japonicus*	4.89
CL2425.Contig1_All	Inhibitor of apoptosis protein	*Penaeus monodon*	1.54
Unigene4922_All	NF-κB transcription factor Relish	*Litopenaeus vannamei*	2.33
CL1572.Contig2_All	Relish	*Litopenaeus vannamei*	1.87
CL2544.Contig1_All	Transcription factor ATF-b	*Litopenaeus vannamei*	2.74
Unigene16368_All	Transcription factor ATF-b	*Litopenaeus vannamei*	6.72
**JAK-STAT signaling pathway**			
CL2469.Contig2_All	bcl-2-like protein 1	*Penaeus vannamei*	1.07
Unigene18396_All	G1/S-specific cyclin-D2-like	*Penaeus vannamei*	−1.18
CL1011.Contig6_All	Protein enhancer of sevenless 2B	*Eurytemora affinis*	4.70
CL1067.Contig2_All	Serine/threonine-protein kinase pim-1-like	*Penaeus vannamei*	2.25
CL1880.Contig2_All	STAT	*Penaeus vannamei*	−0.58
CL470.Contig4_All	Tyrosine-protein phosphatase non-receptor type 11-like	*Penaeus vannamei*	0.81

**FIGURE 8:**
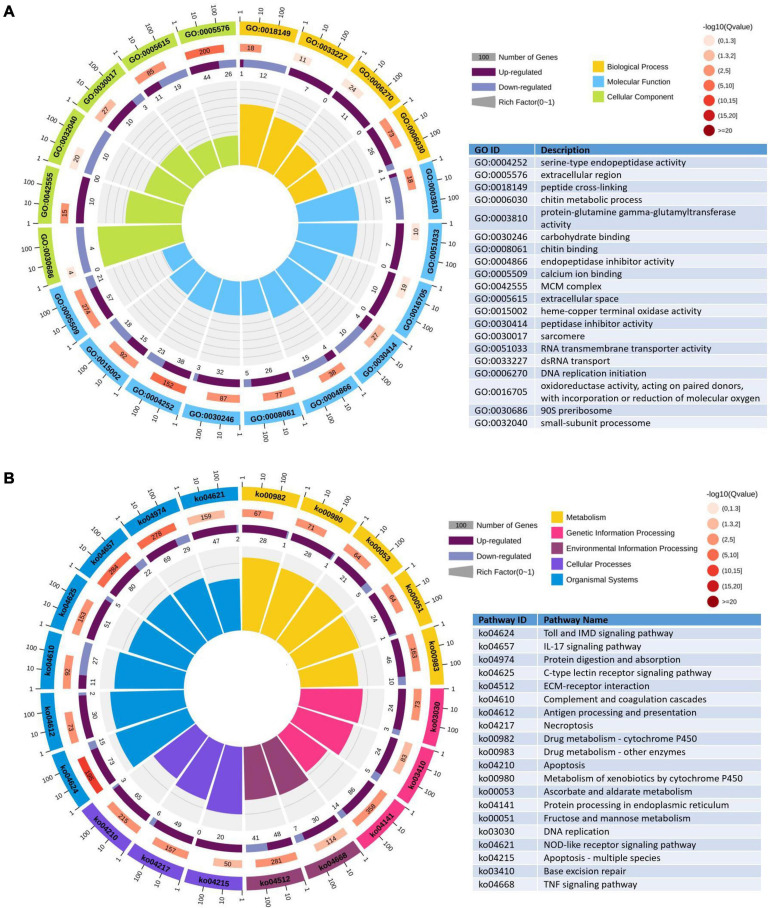
Gene Ontology (GO) terms and Kyoto Encyclopedia of Genes and Genomes (KEGG) pathway enrichment analysis of differentially expressed genes (DEGs). **(A)** The first lap indicates the top 20 GO terms and the number of genes that correspond to the outer lap. The second lap indicates the number of genes in the genome background and the *q*-values for enrichment of the DEGs for the specified biological process. The third lap indicates the ratio of upregulated genes (deep purple) and downregulated genes (light purple). The fourth lap indicates the enrichment factor of each GO term. **(B)** The first lap indicates the top 20 KEGG terms and the number of genes that correspond to the outer lap. The second lap indicates the number of genes in the genome background and *q*-values for enrichment of the DEGs for the specified biological process. The third lap indicates the ratio of upregulated genes (deep purple) and downregulated genes (light purple). The fourth lap indicates the enrichment factor of each KEGG term.

To further evaluate the biological function, all the DEGs were mapped to the term in the GO and KEGG databases. In the GO enrichment analysis, 1,237 upregulated genes and 788 downregulated genes expressed in the DIV1-infected group were divided into three categories with 55 functional groups, consisting of biological progress (24 functional groups), cellular component (19 functional groups), and molecular function (12 functional groups). The top 20 GO terms influenced by DIV1 infection are shown in Figure 8A. Compared to the healthy group, DIV1 infection promoted carbohydrate binding (32 upregulated genes and three downregulated genes), chitin binding (26 upregulated genes and five downregulated genes), chitin metabolic process (26 upregulated genes and four downregulated genes), and DNA replication initiation (11 upregulated genes). These GO terms may be related to virus invasion, replication, and host antiviral infection.

For the KEGG pathway enrichment analysis, 2,140 DEGs were annotated into 249 pathways. Among them, 1,396 upregulated genes were annotated into 247 pathways, and 744 downregulated genes were annotated into 217 pathways. The top 20 KEGG pathway enrichments influenced by DIV1 infection are shown in Figure 8B. It was worth noting that half of them are related to immune response, such as Toll and IMD signaling pathway (73 upregulated genes and 15 downregulated genes), IL-17 signaling pathway (80 upregulated genes and 22 downregulated genes), C-type lectin receptor signaling pathway (51 upregulated genes and five downregulated genes), complement and coagulation cascades (11 upregulated genes and 27 downregulated genes), antigen processing and presentation (30 upregulated genes and two downregulated genes), necroptosis (49 upregulated genes and six downregulated genes), apoptosis (65 upregulated genes and three downregulated genes), NOD-like receptor signaling pathway (47 upregulated genes and two downregulated genes), apoptosis—multiple species (20 upregulated genes), and TNF signaling pathway (30 upregulated genes and seven downregulated genes). Furthermore, almost all of these immune-related pathways were activated after DIV1 infection, except of complement and coagulation cascades.

### Identification of SSR in the Transcriptome

**FIGURE 9:**
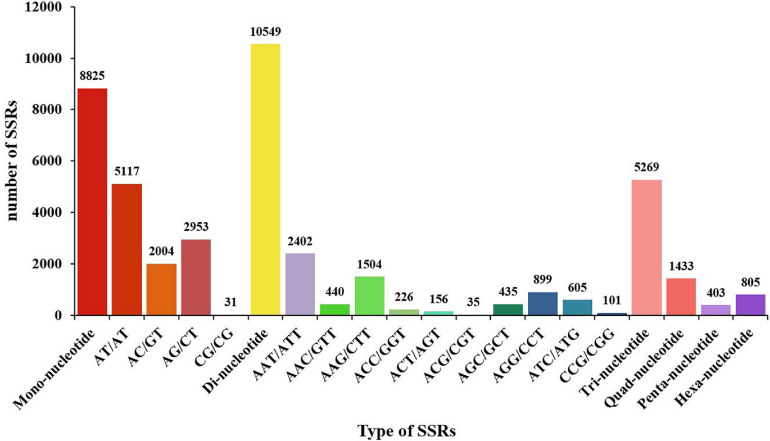
Distribution of SSR nucleotide classes among different nucleotide types found in the transcriptome of *Marsupenaeus japonicus*. The *X*-axis indicates the type of SSRs. The *Y*-axis indicates the number of SSRs.

To date, only a few SSR markers are available for *M. japonicus*. In order to diagnose the disease caused by DIV1 and to lay the foundation for the following genetic breeding studies, SSRs were identified in the *M. japonicus* hemocyte transcriptome. As shown in Figure 9, a total of 20,342 SSRs were obtained, including 8,825 mono-nucleotide repeats, 11,806 di-nucleotide repeats, 5,269 tri-nucleotide repeats, 1,433 quad-nucleotide repeats, 403 penta-nucleotide repeats, and 805 hexa-nucleotide repeats. Among the di-nucleotide repeats, AT/AT (5,117, 18.75%) was the most dominant motif, and the tri-nucleotide repeats was AAT/ATT (2,402, 8.80%) (Figure 9).

### Results of qRT-PCR

**FIGURE 10:**
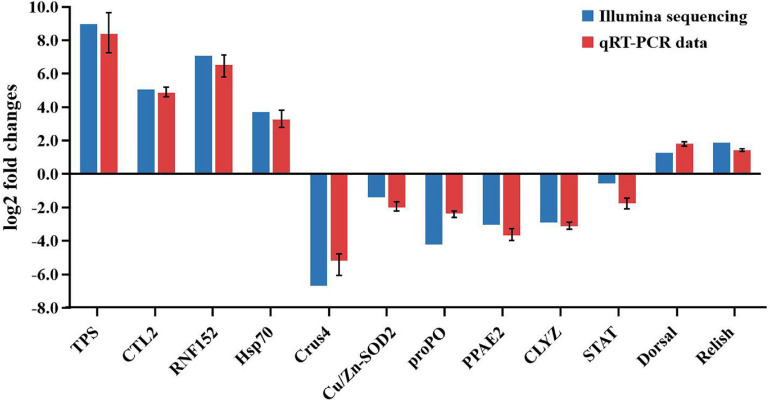
Comparison of the expression profiles of 12 selected genes as determined by Illumina sequencing and qRT-PCR.

To validate the sequencing results, five upregulated genes and seven downregulated genes were chosen for the qRT-PCR analysis, including trypsin (TPS), C-type lectin 2 (CTL2), E3 ubiquitin-protein ligase RNF152-like (RNF152), HSP70, prophenoloxidase (proPO), crustin-like peptide type 4 (Crus4), copper/zinc superoxide dismutase isoform 5 (Cu/Zn-SODi5), PPAE2, C-type lysozyme (CLYZ), STAT, Dorsal, and Relish. These genes are all related to immunity. As shown in Figure 10, the expression patterns of these tested genes were consistent when the two different methods were used. The result proved that the gene expression profiles derived from RNA-seq were reliable and confirmed the expression changes of these genes in response to DIV1 infection. The transcriptome and qRT-PCR results showed that, in response to DIV1 infection, the activation of the Toll and IMD signaling pathways results in the upregulated activities of the transcription factors Dorsal and Relish. On the contrary, the expression of STAT was downregulated under DIV1 infection, which affected the role of the JAK-STAT signaling pathway in resisting viral infection (Figure 10).

## Discussion

As a new type of virus, DIV1 has brought a huge challenge to the shrimp farming industry because of its lethal and virulent. Up to now, it has been found that DIV1 can be successfully infected in many species of shrimp, and transcriptome analysis has been performed on *L*. *vannamei*, *P*. *monodon*, *F*. *merguiensis*, and *C. quadricarinatus* infected with DIV1 (Liao X. et al., 2020; Liao X. Z. et al., 2020; Yang H. et al., 2020; He et al., 2021). These studies had found from the transcriptome level that DIV1 infection can cause a strong immune response in shrimps and found that some immune pathways and genes play an important role in the process of resisting DIV1 infection. *M. japonicus* is one of the most widely distributed marine shrimp species. However, there are no detailed reports and transcriptome studies on the DIV1 infection of *M. japonicus*. In this study, we proved that *M. japonicus* is the host of DIV1 by artificial injection and measured the toxicity of DIV1 to *M. japonicus* by the LC_50_ test. Enzyme activity test can reflect the impact of DIV1 infection on the immunity of *M. japonicus*, and the hemocyte transcriptome analysis of *M. japonicus* infected with DIV1 was helping to better understand the hemocyte immune mechanism of *M. japonicus* against DIV1 infection.

Through the method of artificial injection of DIV1, it was determined that *M. japonicus* could be a host of DIV1. The DIV1-infected *M. japonicus* showed obvious symptoms of empty stomach and intestine, yellow hepatopancreas, red body, and soft shell. In previous studies, it was also found that, after infection with DIV1, all *L. vannamei*, *M. rosenbergii*, *E. carinicauda*, and *P*. *monodon* had symptoms of empty stomach and intestine, atrophy of the hepatopancreas with fading color, and soft shell (Chen X. et al., 2019; Qiu et al., 2019; Liao X. et al., 2020; He et al., 2021). These symptoms can be considered as the basic symptoms of shrimp infection with DIV1. In addition, different species of shrimps infected with DIV1 seem to have some different characteristics. Some of the DIV1-infected *L*. *vannamei* and *P*. *monodon* showed a black edge of the abdominal shell (Liao X. et al., 2020; He et al., 2021), while some of the DIV1-infected *M. rosenbergii* were accompanied by slightly whitish muscle and mutilate antenna (Qiu et al., 2019). All of the DIV1-infected *P*. *monodon* body color turned black while all of the DIV1-infected *M. japonicus* body color turned red (He et al., 2021). All of the DIV1-infected *M. rosenbergii* had a distinct white triangle area under the carapace at the base of the frontal angle, while the hepatopancreas tissue at the base of the rostrum of all the DIV1-infected *E. carinicauda* had a slight cloudy white shade (Chen X. et al., 2019; Qiu et al., 2019). Not surprisingly, the LC_50_ results of DIV1 in *M. japonicus* was different from that in *L*. *vannamei* and *P*. *monodon* (Liao X. et al., 2020; He et al., 2021). This difference is caused by different species, individual size, and the quality of the aquaculture water. These comprehensive symptoms will help us to initially judge whether DIV1 infection has occurred during shrimp farming, and the LC_50_ test results provided reference for constructing a *M. japonicus*-DIV1 infection model.

The results of the enzyme activity test found that the activities of the four immune enzymes were generally inhibited after DIV1 infection at 24 h. Oxidative burst is an important immune mechanism in shrimp. The shrimp hemocytes will produce a large amount of ROS during phagocytosis, which can effectively eliminate foreign harmful substances and pathogens, such as invading bacteria and viruses (Xian et al., 2017). However, excessive ROS can cause oxidative damage to the organism. SOD and CAT can effectively remove ROS. Under the action of SOD, ROS react with hydrogen ions to generate hydrogen peroxide and then react with hydrogen ions under the action of CAT to finally produce harmless water. After *M. japonicus* was infected with DIV1, the activities of SOD (*p* < 0.001) and CAT (*p* < 0.05) in the plasma were decreased significantly. It meant that the ability of *M. japonicus* to produce and remove ROS was unbalanced, and the excess ROS caused damage to the tissues and organs of the shrimp. The activities of LYZ and PO can directly reflect the strength of the immunity of the host. LYZ is widely distributed in the animal kingdom. It catalyzes the hydrolysis of bacterial cell walls and acts as a non-specific innate immunity factor against the invasion of bacterial pathogens (Jollès and Jollès, 1984). Some recent studies found that LYZ also shows antiviral activity against WSSV and IHHNV in shrimp (Mai and Wang, 2010; Cai et al., 2019). The melanization pathway activated by the proPO system is a principal innate immune response in shrimp (Amparyup et al., 2013; Charoensapsri et al., 2014). Upon pathogen invasion, proPO is activated by the proteolytic cleavage into the active PO, which leads to the initiation of melanin formation (Tassanakajon et al., 2017). In our study, DIV1 infection caused a significant decrease in LYZ (*p* < 0.001) and PO activity (*p* < 0.01), which meant that DIV1 inhibit the immune function of the host to a certain extent.

Through the RNA sequencing platform, a total of 52,287 unigenes were *de novo* assembled, and 20,342 SSR markers associated with these unigenes were obtained. Through a comparative transcriptomic analysis, 6,900 DEGs (including 3,882 upregulated genes and 3,018 downregulated genes) were screened in the DIV1-infected group and the negative control group, with a cut-off *q*-value <0.05 and |log2 (fold change)| > 1. These included a variety of immune enzyme-related genes, such as PPAE2, proPO, Cu/Zn-SODi5, and CLYZ. It was worth noting that the expression of all these four immune enzyme-related DEGs decreased significantly after DIV1 infection, which had the same trend as the qRT-PCR analysis and the enzyme activity test. These three different methods all proved that DIV1 infection suppressed the immune ability of *M. japonicus*. In order to validate the sequencing results, in addition to the four genes mentioned above, another five DEGs were selected to perform qRT-PCR, including TPS, CTL2, RNF152, HSP70, and Crus4. The results showed that the gene expression profiles derived from RNA-seq were reliable. HSP70 had been found to take part in the innate immune response of *L. vannamei* against WSSV and IHHNV (Valentim-Neto et al., 2014; Janewanthanakul et al., 2019). In one of our previous studies, HSP70 in *P. monodon* was significantly upregulated under DIV1 challenges (He et al., 2021). Similar to the previous study, HSP70 in *M. japonicus* was also significantly upregulated after DIV1 infection, which meant that HSP70 plays an important role in anti-viral infection. Crustin is an antimicrobial peptide (AMP) that plays a key role in the innate immunity of crustaceans (Li M. et al., 2019). In present study, DIV1 infection caused a significant downregulation of Crus4 in *M. japonicus*. This result was the same with that of our previous study in the sense that DIV1 infection can inhibit the expression level of Crus4 and Crus1 in *P. monodon* (He et al., 2021). These results suggest that DIV1 can achieve immune escape by suppressing the expression of Crustin.

To further understand the response of the hemocytes under DIV1 infection, all the DEGs were mapped to the terms in the GO and KEGG databases. The results showed that some GO terms related to virus invasion, replication, and host antiviral infection were promoted under DIV1 infection, such as carbohydrate binding, chitin binding, chitin metabolic process, and DNA replication initiation. Lectins are a kind of carbohydrate-binding proteins, which play roles in various biological processes, including immune response, cell adhesion, and glycoprotein metabolism (Wang et al., 2020). In the DIV1-infected *L. vannamei*, *F. merguiensis*, and *C. quadricarinatus*, the expression of C-type lectin was significantly upregulated (Yang H. et al., 2020; Liao X. et al., 2020). This phenomenon was also found in our study; some C-type lectins such as CTL1 and CTL2 were significantly upregulated after DIV1 infection. Several studies had shown that shrimp C-type lectin can bind to several structural proteins of WSSV, thereby inhibiting WSSV gene expression or reducing host mortality caused by the WSSV infection (Zhao et al., 2009; Xu et al., 2013; Kwankaew et al., 2017; Phanthipha et al., 2018). However, some shrimp C-type lectins had apparently opposite functions during viral infection. It had been found that WSSV and yellow head virus (YHV) can hijack the shrimp C-type lectin for successful adhesion and entry into the host cell (Junkunlo et al., 2012; Wang et al., 2014; He et al., 2015). These different research results reflect the functional diversity and complexity of shrimp C-type lectins. The roles of these C-type lectins in the process of DIV1 infection in *M. japonicus* needs further study. Previous studies showed that chitin-binding proteins can facilitate WSSV gene expression and genome replication (Li et al., 2015; Yang et al., 2018; Yang F. et al., 2020; Chen et al., 2021). Here the GO terms related to chitin and DNA replication were upregulated after DIV1 infection, including chitin binding, chitin metabolic process, and DNA replication initiation. This implied that the protein containing the chitin-binding domain was involved in the invasion and replication process of DIV1.

Among the top 20 KEGG pathway enrichments influenced by DIV1 infection, half of them were found to be immune-related pathways, including the Toll and IMD signaling pathway, IL-17 signaling pathway, C-type lectin receptor signaling pathway, complement and coagulation cascades, antigen processing and presentation, necroptosis, apoptosis, NOD-like receptor signaling pathway, apoptosis—multiple species, and TNF signaling pathway. It was worth noting that almost all of these immune-related pathways were promoted after DIV1 infection, except for complement and coagulation cascades. The results reflect the fierce immune response of hemocytes during DIV1 attack. Similar to the results of the GO enrichment, the enrichment of C-type lectin receptor signaling pathway also meant that C-type lectins play a very important role in DIV1 infection. Through the analysis of the immune-related pathways, it was found that caspase-related genes were related to a variety of immune-related pathways, such as the Toll and IMD signaling pathway, IL-17 signaling pathway, C-type lectin receptor signaling pathway, necroptosis, apoptosis, NOD-like receptor signaling pathway, apoptosis—multiple species, and TNF signaling pathway. Apoptosis is characterized by a number of characteristic morphological changes in the structure of the cell (Hengartner, 2000), and caspase (cysteinyl aspartate specific proteinase) is an important protein family for the procedure of apoptosis (Fan et al., 2010). Caspase-induced apoptosis shows two sides in the process of shrimp virus infection. On the one hand, some caspase-induced shrimp cell apoptosis is a protective antiviral response—for example, the upregulated caspase 1 and caspase 3 can enhance the apoptotic activity and are involved in the innate immunity of shrimp to inhibit the WSSV replication (Chen Y. et al., 2019; Yang et al., 2019). On the other hand, the caspase-induced kakoapoptosis under viral infection can lead to massive apoptosis and finally cause the death of the infected shrimp (Flegel and Sritunyalucksana, 2011)—for example, after shrimp were infected with WSSV or YHV, the number of apoptotic cells increased with the degree of infection, and vital tissues such as the hemolymph, gills, heart, and lymphoid organs had been severely compromised (Sahtout et al., 2001; Khanobdee et al., 2002). Several recent studies found that caspase and caspase 4 of *F. merguiensis*, caspase 2 of *L. vannamei* and caspase 1 of *P. monodon* were all up-regulated in the hemocytes after DIV1 infection (Liao X. et al., 2020; Liao X. Z. et al., 2020; He et al., 2021). This similar situation also occurred in the present study, that is, all caspase-related genes were significantly upregulated after *M. japonicus* was infected with DIV1 (Table 3). It was especially worth noting that DEGs were significantly enriched in necroptosis after DIV1 infection. Necroptosis and apoptosis are two processes of cell death (Bertheloot et al., 2021). Differently from apoptosis, necroptosis is generally characterized to be the uncontrolled death of the cell, usually following a severe insult, resulting in spillage of the contents of the cell into surrounding tissues and subsequent damage thereof (D’Arcy, 2019). These results indicated that DIV1 has great toxicity and damage to host cells, and apoptosis and necroptosis may be the ultimate causes of shrimp death. The JAK-STAT, Toll, and IMD signaling pathways are considered the main pathways to regulate the innate immune response in shrimp (Li and Xiang, 2013; Li M. et al., 2019). STAT is one of the main cellular components in the JAK-STAT signaling pathway, Dorsal is the critical transcription factor in the Toll signaling pathway, and Relish plays a key role in the IMD signaling pathway (Li et al., 2009; Agaisse and Perrimon, 2010; Huang et al., 2016). WSSV infection can activate STAT in shrimp, although it is still controversial whether this activation promotes or inhibits WSSV infection (Chen et al., 2008; Sun et al., 2011; Ren et al., 2015). In the present study, DIV1 infection significantly inhibited the expression of STAT. The effect of DIV1 infection on the JAK-STAT signaling pathway of *M. japonicus* needs further study. A previous review concluded that WSSV infection activates the NF-κB-related signaling pathways (Toll and IMD signaling pathway), which stimulates the transcription factors including Dorsal and Relish, resulting in the expression of several sets of effectors such as AMPs (Li et al., 2018). In this study, the expression of Dorsal and Relish was significantly upregulated after DIV1 infection. It meant that the Toll and IMD signaling pathways play key roles in *M. japonicus* immune response to DIV1. A meta-analysis of the four reported shrimp hemocyte transcriptomes may help to further reveal the potential therapeutic targets of DIV1.

## Conclusion

In conclusion, enzyme activity study and transcriptomics analysis showed that DIV1 had an inhibitory effect on the immune enzyme activity of shrimp. The immune-related signaling pathways in hemocytes were significantly activated under DIV1 challenge, and HSP70, C-type lectins, and caspase may play an important role against DIV1 infection.

## Data Availability Statement

The datasets presented in this study can be found in online repositories. The names of the repository/repositories and accession number(s) can be found below: https://www.ncbi.nlm.nih.gov/, PRJNA720475.

## Ethics Statement

The study protocol was approved by the Ethics Review Board of the Institutional Animal Care and Use Committee in Guangdong Ocean University. Written informed consent was obtained from the owners for the participation of their animals in this study.

## Author Contributions

ZH, CS, and SZ contributed to conception and design of the study. ZH, JcZ, XC, ML, YX, JnZ, HC, and GC collected the samples and performed the experiments. ZH organized the database and wrote the first draft of the manuscript. SZ performed the writing – review and editing. CS contributed to the project administration and funding acquisition. All authors contributed to manuscript revision, read, and approved the submitted version.

## Conflict of Interest

GC was employed by company Haimao Seed Technology Group Co., Ltd. The remaining authors declare that the research was conducted in the absence of any commercial or financial relationships that could be construed as a potential conflict of interest.

## Publisher’s Note

All claims expressed in this article are solely those of the authors and do not necessarily represent those of their affiliated organizations, or those of the publisher, the editors and the reviewers. Any product that may be evaluated in this article, or claim that may be made by its manufacturer, is not guaranteed or endorsed by the publisher.

## Footnotes

1
http://www.genome.jp/kegg

2
http://geneontology.org

3
ftp://ftp.ncbi.nlm.nih.gov/blast/db

4
http://www.uniprot.org/

5
https://www.ncbi.nlm.nih.gov/COG/

6
http://pgrc.ipk-gatersleben.de/misa/

7
https://www.ncbi.nlm.nih.gov/sra
